# Exosomal miR-331-3p derived from chemoresistant osteosarcoma cells induces chemoresistance through autophagy

**DOI:** 10.1186/s13018-023-04338-8

**Published:** 2023-11-22

**Authors:** Chenyang Meng, Yun Yang, Wei Feng, Penglei Ma, Rui Bai

**Affiliations:** 1 Department of Orthopedic Surgery, Second Affiliated Hospital of Inner Mongolia Medical University, Hohhot, China; 2 Department of Anesthesiology, Second Affiliated Hospital of Inner Mongolia Medical University, Hohhot, China

**Keywords:** Osteosarcoma, Exosome, microRNA-331, Autophagy, Chemosensitivity, Chemoresistance

## Abstract

**Background:**

Osteosarcoma is a common malignant bone tumor, and chemotherapy can effectively improve the prognosis. MicroRNA-331 (MiR-331) is associated with poor cancer outcomes. However, the role of miR-331 in osteosarcoma remains to be explored.

**Methods:**

Drug-resistant osteosarcoma cells were cultured, and their exosomes were purified. The secretion and uptake of exosomes by drug-resistant osteosarcoma and osteosarcoma cells were confirmed using a fluorescence tracking assay and Transwell experiments. The effects of drug-resistant exosomes on cell proliferation were determined using a 3-(4,5-dimethylthiazol-2-yl)-2,5-diphenyltetrazolium bromide assay. siRNA-Drosha and neutral sphingomyelinase inhibitor GW4869 were used to determine the transfer of miRNAs. qRT-PCR and western blotting were used to detect the role of autophagy in the regulation of drug-resistant cell-derived exosomal miR-331-3p.

**Results:**

Exosomal miR-331-3p levels in drug-resistant cells were higher than in exosomes from osteosarcoma cells. The exosomes secreted by the drug-resistant osteosarcoma cells could be absorbed by osteosarcoma cells, leading to acquired drug resistance in previously non-resistance cells. Inhibition of miRNAs resulted in reduced transmission of drug resistance transmission by exosomes. Exosomes from drug-resistant osteosarcoma cells transfected with siRNA-Drosha or treated by GW4869 could not enhance the proliferation of MG63 and HOS cells. Finally, miR-331-3p in the exosomes secreted by drug-resistant osteosarcoma cells could induce autophagy of osteosarcoma cells, allowing them to acquire drug resistance. The inhibition of miR-331-3p decreased drug resistance of osteosarcoma cells.

**Conclusion:**

Exosomes secreted from chemoresistant osteosarcoma cells promote drug resistance through miR-331-3p and autophagy. Inhibition of miR-331-3p could be used to alleviate drug resistance in osteosarcoma.

## Background

Osteosarcoma is a rare tumor of mesenchymal origin characterized by the production of osteoid (immature bone) by malignant cells [1–3], mainly in individuals > 60 years old and between 10 and 19 years [2–4]. Osteosarcoma metastasizes fast and has a high mortality rate [1–4]. The relapse rate in patients with osteosarcoma is 30% with localized disease and 80% with metastatic disease, and the long-term survival in patients with recurrent disease is < 20% [3]. Chemotherapy (mostly based on methotrexate, doxorubicin, and cisplatin (CDDP)) can improve the survival of osteosarcoma patients combined with surgery [5, 6], but chemoresistance can affect the outcome of osteosarcoma [7]. Therefore, exploring the mechanism of chemotherapy resistance in osteosarcoma is very important.

Exosomes (exo) play important roles in the transport of material (e.g., proteins, metabolites, and RNA) between cells [8]. Active tumor cells secrete more exo into the tumor microenvironment than normal cells or dormant tumor cells would [9]. Tumor cell-secreted exo can transform normal cells into tumor cells [9]. Besides, exo contain bioactive molecules (including microRNAs (miRNAs) [10]) and cytokines that regulate the signaling pathway of the recipient cells, further regulating the invasion, proliferation, and chemosensitivity of the tumor cells [11]. miRNAs can target the 3’ untranslated region of their target mRNAs directly, suppressing mRNA translation into protein [12], playing significant roles in multiple cellular pathways, and can regulate tumor cell invasion, proliferation, and chemosensitivity [13–15]. Bone marrow mesenchymal stem cells (BMSCs)-derived exo can regulate osteosarcoma proliferation through the Hedgehog signaling pathway [16]. In addition, drug-resistant osteosarcoma cells can transfer multiple drug-resistant phenotypes to non-drug-resistant cells via exo, thereby inducing drug resistance in previously non-drug-resistant osteosarcoma cells [17]. It has been reported that miR-331-3p might lead to poor cancer outcomes in breast cancer [18], but there are no studies on the association between miR-331-3p and osteosarcoma.

Autophagy is markedly associated with drug resistance in osteosarcoma [19]. In autophagy, damaged organelles, mitochondria, and macromolecules are degraded, which plays important roles in cell growth, development, differentiation, and death [20]. Various chemotherapy drugs can induce autophagy, and autophagy is correlated with drug resistance [19, 21, 22]. Several chemotherapy drugs induce apoptosis in cancer cells, but the cancer cells can upregulate autophagy to escape apoptosis [19].

Therefore, this study aimed to investigate the relationship between exo miR-331-3p and osteosarcoma and to explore whether miR-331-3p could regulate drug resistance via autophagy in osteosarcoma. The results could provide a better understanding of the role of miRNAs in osteosarcoma and the implications in clinical practice.

## Methods

### Osteosarcoma cells culture

The NHOst cell line (PX-2538RL, Lonza, USA) and osteosarcoma cell (OSC) lines MG63 (#CL-0157), HOS (#CL-0360), U2OS (#CL-0236), and Saos2 (#CL-0202) (OSCs were all from Procell Life Science & Technology, Co., Ltd., Wuhan, China) were purchased and cultured in minimal essential medium (MEM, PM150410, Pricella, Wuhan, China) containing 10% fetal bovine serum, 100 U/mL penicillin, and 100 μg/mL streptomycin in a cell incubator at 37 ℃ and 5% CO_2_. The cells were re-suspended in 3 mL of medium and then subcultured in a petri dish at a 1:3 dilution.

### Culture of cisplatin-resistant OSCs

CDDP-resistant OSCs were induced as previously described [23]. When the MG63, HOS, U2OS, and Saos2 cells grew to the logarithmic phase, 0.1 mg/L CDDP (MB1055, Meilune, Dalian, China) was added to the culture medium. After 24 h, the culture medium was replaced with a medium lacking CDDP. Cell passage was performed after the stable growth of the cells. This method was repeated five times. The CDDP concentration was increased to 0.2 mg/L for a further five cycles and then to 0.5 mg/L for three cycles. After a total of 190 days of induction, OSCs could grow stably and passage normally in the presence of 0.5 mg/L CDDP, indicating that the cell line could tolerate 0.5 mg/L CDDP. The drug-resistant strain was named OSC/CDDP. After overnight cell culture, the medium was replaced with drug-containing medium at various concentrations: 0, 0.25 μg/mL, 0.5 μg/mL, 1 μg/mL, 2 μg/mL, 4 μg/mL, 8 μg/mL, 16 μg/mL, 32 μg/mL, and 64 μg/mL. The cells were then incubated at 37 degrees Celsius with 5% CO_2_ for 72 h. MTT assay was performed, and the IC50 was calculated.

### Transfection of miR-331 inhibitor

Prepared sufficient 1.5 mL EP tubes and mixed as follows: added 10 μL of Exo-Fect solution, 20 μL of miRNA inhibitor (20 μmol of miR-331-3p inhibitor, miR-NC inhibitor, Genepharma, Shanghai), 70 μL of sterile 1 × PBS, and 50 μL of sterile 1 × PBS re-suspended MG63/CDDP extracellular vesicles, totaling 150 μL of transfection reaction system. Placed the EP tubes in a 37 ℃ mixer and incubated for 10 min, then immediately placed on ice. Added 30 μL of ExoQuick-TC (EXFT10A-1, SBI, USA) provided in the kit to the transfection reaction system and inverted 6 times to mix. Placed the EP tubes on ice (or 4 ℃) and incubated for 30 min. Centrifuged at 13,000 rpm (5417R, Eppendorf, USA) for 3 min. Discarded the supernatant and rsuspended the precipitated transfected MG63/CDDP extracellular vesicles in 300 μL of 1 × PBS. Collected the transfected MG63/CDDP extracellular vesicles.

### Quantitative real-time reverse transcription-polymerase chain reaction (qRT-PCR)

A Direct Zol RNA microPrep kit (Zymo Research, Irvine, CA, USA) was used to extract the intracellular RNA and exo RNA. cDNA was synthesized using the PrimScript RT reagent kit with gDNA Eraser (#RR047A, Takara, Otsu, Japan) and the SYBR Premix Ex Taq (#RR820A, Takara, Otsu, Japan) from 200 ng of isolated RNA. The cDNA was used as the template in qRT-PCR reactions performed on an ABI Prism 7500 sequence detection system (Applied Biosystems, Foster City, CA, USA). The sequences of primers are shown in Table 1. *E*_target_ was calculated as the amplification efficiency of the target gene, *E*_ref_ was the amplification efficiency of the reference gene, △and Ct was the difference between the Ct value of the control group and the sample. The formula is as follows:

$${\text{ratio}} = \frac{{(1 + E_{{{\text{target}}}} )^{{\Delta {\text{Ct}}_{{{\text{target}}}} \left( {{\text{control}} - {\text{sample}}} \right)}} }}{{(1 + E_{{{\text{ref}}}} )^{{\Delta {\text{Ct}}_{{{\text{ref}}}} \left( {{\text{control}} - {\text{sample}}} \right)}} }}$$

**Table 1:** Primers used for qRT-PCR

Name	Sequence (5′- > 3′)	
miR-331-3p	F	CACAACTCGAGAACGTACAGAAGGCTCCAGAAATG
	R	TGAAGATCTGAAGGATTAACCAACCAATTTTTGC
miR-199a	F	ACACTCCAGCTGGGCCCAGTGTTCAGACTAC
	R	TGGTGTCGTGGAGTCG
U6	F	CTCGCTTCGGCAGCACA
	R	AACGCTTCACGAATTTGCGT

### Cell transfection

At 24 h before OSC transfection, the cells were digested with trypsin, their concentration was adjusted to 2 × 10^5^ cells/mL, and the cells were inoculated into 6-well culture plates. After the cells adhered to the wells, they were cultured in 2 mL of Dulbecco’s modified Eagle’s medium (DMEM, PM150210, Pricella, Wuhan, China) containing serum but without antibiotics. For transfection, the mimics (miR-199a, GenePharma, Shanghai, China) were diluted with 200 μL Opti-MEM medium, mixed gently, and kept for 5 min at room temperature. At the same time, 5 μL of Lipofectamine 2000 was diluted with 200 μL of Opti-Mem I medium, mixed gently, and kept for 5 min at room temperature. The diluted mimics and Lipofectamine 2000 were mixed, kept for 25 min at room temperature, added into the cell culture well, and shaken to mix evenly. At 48 h after transfection, the expression level of miR-199a in the cells was detected using qRT-PCR.

### Extraction and identification of exosome

OSCs were grown to 70–75% confluence, the original culture medium was discarded, and the cells were washed with PBS three times. Serum-free medium was added for further culture for 36–48 h. The culture medium was collected and centrifuged at 4 °C at 300 ×*g* for 10 min to remove the remaining cells, at 4 °C for 20 min at 2000 ×*g* to remove the cell fragments, and at 4 °C for 45 min at 11,000 ×*g* to remove the impurities. The supernatant was retained and then ultracentrifuged at 110,000 ×*g* at 4 ℃ for 90 min, and the supernatant was discarded. One milliliter of 1 × PBS was added to each centrifuge tube to resuspend the precipitate, and the suspension was transferred to a new ultracentrifuge tube and ultracentrifuged at 110,000 ×*g* at 4℃ for 70 min. The supernatant was discarded, and the precipitate (containing exo) was re-suspended in 100 μL of 1 × PBS. Transmission electron microscopy (JEM-2100, JEOL Ltd., Tokyo, Japan) of exo and granulometric analysis (ZETASIZER Nano series-Nano-ZS; Malvern Analytical, Malvern, UK) were performed. Exo were stained using anti-CD63 (#ab18235, Abcam, Cambridge, United Kingdom) and anti-CD81 (#ab239256, Abcam, Cambridge, UK) antibodies. The antibody dilution concentration was 1:1000. Flow cytometry (Accuri C6 flow cytomenter and BD FACSDiva, BD Biosciences, USA) was performed according to the instrument operation procedure.

### PKH26-labeled exosomes

Exo (100 µL of an exo solution at 100 μg/mL) were suspended in 1 mL of PBS, and 4 μL of PKH26 fluorescent dye solution was added and incubated at 37 ℃ for 20 min. The solution was ultracentrifuged at 100,000 ×*g* for 70 min, and the supernatant was discarded. The exo were re-suspended in 10 mL of PBS and ultracentrifuged at 100,000 ×*g* at 4 ℃ for 70 min. The excess dye was removed, the supernatant was discarded, and the exo were suspended in 100 μL of PBS. The PKH26-labeled exo and OSCs were cultured together in MEM medium containing 10% fetal bovine serum, 100 U/mL penicillin, and 100 µg/mL streptomycin in a cell incubator at 37℃ and 5% CO_2_ for 12 h. The medium was removed, and the cells were washed twice with PBS, fixed with 4% paraformaldehyde, and stained with 4′,6-diamidino-2-phenylindole (DAPI). The cells stained red by PKH26 were observed under a confocal fluorescence microscope.

### Transwell assay

Cy3-miR-199a mimic was transfected into OSC/CDDP cells. Then, OSC/CDDPs (1 × 10^6^/well) and OSCs were co-cultured at a 1:1 ratio on a Transwell plate for 12 h. The upper compartment contained OSC/CDDPs, and the lower compartment contained OSCs. In the control group, only Cy3 was transfected without miR-199a. After rinsing the cells twice with PBS, the fluorescence of Cy3 in the OSCs was observed under a confocal microscope.

### Verification of exosome function

Exo were extracted from drug-resistant OSC/CDDPs and from non-drug resistant. The OSCs were divided into three groups: the OSC treated with drug-resistant exo group (Exo/CDDP), the OSC treated with non-drug-resistant exo group (Exo/S), and the OSC treated with PBC blank control group (PBS + OSC). Exo (from drug-resistant or non-drug-resistant cells) (2 μg) were added to OSCs (1 × 10^6^/well) to establish the experimental group. After co-culture for 12 h, CDDP (1, 10, 30, and 60 ng/mL) was added to the three groups, respectively, for 24 h, and then the cell activity was detected using a 3-(4,5-dimethylthiazol-2-yl)-2,5-diphenyltetrazolium bromide (MTT) assay.

### MTT assay

After CDDP treatment, 10 μL of MTT (Biosharp, Shanghai, China) was added to the cells in each well for 3 h. The α-MEM was removed from the wells, and 150 μL dimethyl sulfoxide was added to each well and shaken evenly. The absorbance of each well was measured at 570 nm using a Multiskan 51,119,000 microplate reader (Thermo Fisher Scientific, Waltham, MA, USA).

### Flow cytometry assay

CDDP (2 μM) was added to the OSC + PBS, Exo/S, Exo/CDDP, and OSC/CDDP cell groups. After incubation for 24 h, flow cytometry was performed. Triton X-100 (0.1%) was added to break the membrane of the cells, which were then centrifuged at 1000 rpm for 5 min. The cells were re-suspended in PBS and centrifuged at 1000 rpm for 5 min. An anti-microtubule-associated protein 1 light chain 3 alpha (LC3) antibody (#ab63817, Abcam, Cambridge, United Kingdom) was added (1:500, dissolved in 1% bovine serum albumin (BSA)) and incubated at room temperature for 2 h. After centrifugation at 1000 rpm for 5 min, the cells were re-suspended in PBS and centrifuged again at 1000 rpm for 5 min. In the dark, the goat anti-rabbit HRP-conjugated secondary antibody (1:2000, dissolved in 1% BSA, #ab6721, Abcam, Cambridge, United Kingdom) was added and incubated at room temperature for 1 h. The cells were centrifuged at 1000 rpm for 5 min, re-suspended in PBS, centrifuged again, and suspended in PBS for flow cytometry detection (Accuri C6 system, BD Biosciences, Franklin Lake, NJ, USA).

### Verification of the functional miRNAs in exosomes

Since there were no expression differences of miR-199a between OSCs and OSCs/CDDP, miR-199a was used to identify the transmission of miRNA between OSC/CDDP and OSC. The cells were divided into three groups: OSC/CDDP + OSC (G1), OSC + OSC/CDDP + GW4869 (10 μM, cultured for 24 h; G2), and OSC (G3). In groups G1 and G2, the OSCs and OSCs/CDDP were co-cultured (1 × 10^6^/well for OSC and OSC/CDDP), and the expression of miR-199a was detected using qRT-PCR. Then, by knocking out the Drosha expression of OSC/CDDP using a small interfering RNA (siRNA-Drosha), the activity of exo-miRNA was inhibited. The levels of miR-331-3p and miR-199a in exo were detected by qRT-PCR to determine whether miRNA loading into exo was inhibited by knocking out the Drosha protein. Then, the exo of OSC/CDDP were extracted and co-cultured with OSCs. The exo were divided into three groups: OSC + Exo/CDDP (treated with siRNA-Drosha), OSC + Exo/CDDP (not treated with siRNA-Drosha), and OSC + PBS. After 12 h of culture, 2 μ CDDP was added to each well, and the cells were subjected to MTT detection.

### Western blotting

Cells or exo were lysed on ice using a radioimmunoprecipitation assay buffer containing protease inhibitors. Equal amounts of proteins from the different groups were separated using 10% sodium dodecyl sulfate–polyacrylamide gel electrophoresis and transferred onto a 0.22-μm polyethylenedifluoride membrane. The membrane was incubated in 5% skim milk at room temperature for 1 h and then with primary antibodies at 4 ℃ overnight: LC3 (#ab63817, Abcam, Cambridge, United Kingdom), p62 (#ab91526, Abcam, Cambridge, United Kingdom), and β-actin (#ab8227, Abcam, Cambridge, United Kingdom). The next day, the membrane was washed with Tris-buffered saline-Tween 20 (TBST) and incubated with the goat anti-rabbit HRP-conjugated secondary antibody (#ab6721, Abcam, Cambridge, United Kingdom). The antibody dilution concentration was 1:1000. Immunoreactive protein bands were detected using an enhanced chemiluminescence kit. Photographs were taken using the Tanon 5200 chemiluminescence imaging system (Tanon, USA) and used ImageJ software (1.52a, NIH, USA) for analysis.

### Statistical analysis

Each experiment was performed three times, and the results are shown as means ± standard deviations. Prism (8.0.2 Version, GraphPad Software, CA, USA) was used for statistical analysis. Differences among three or more groups were compared using a one‑way analysis of variance (ANOVA) and Tukey’s post hoc test. Two-sided *P* values < 0.05 were considered statistically significant.

## Results

### The expression of miR-331-3p was higher in the MG63 and HOS cell lines

**Fig. 1:**
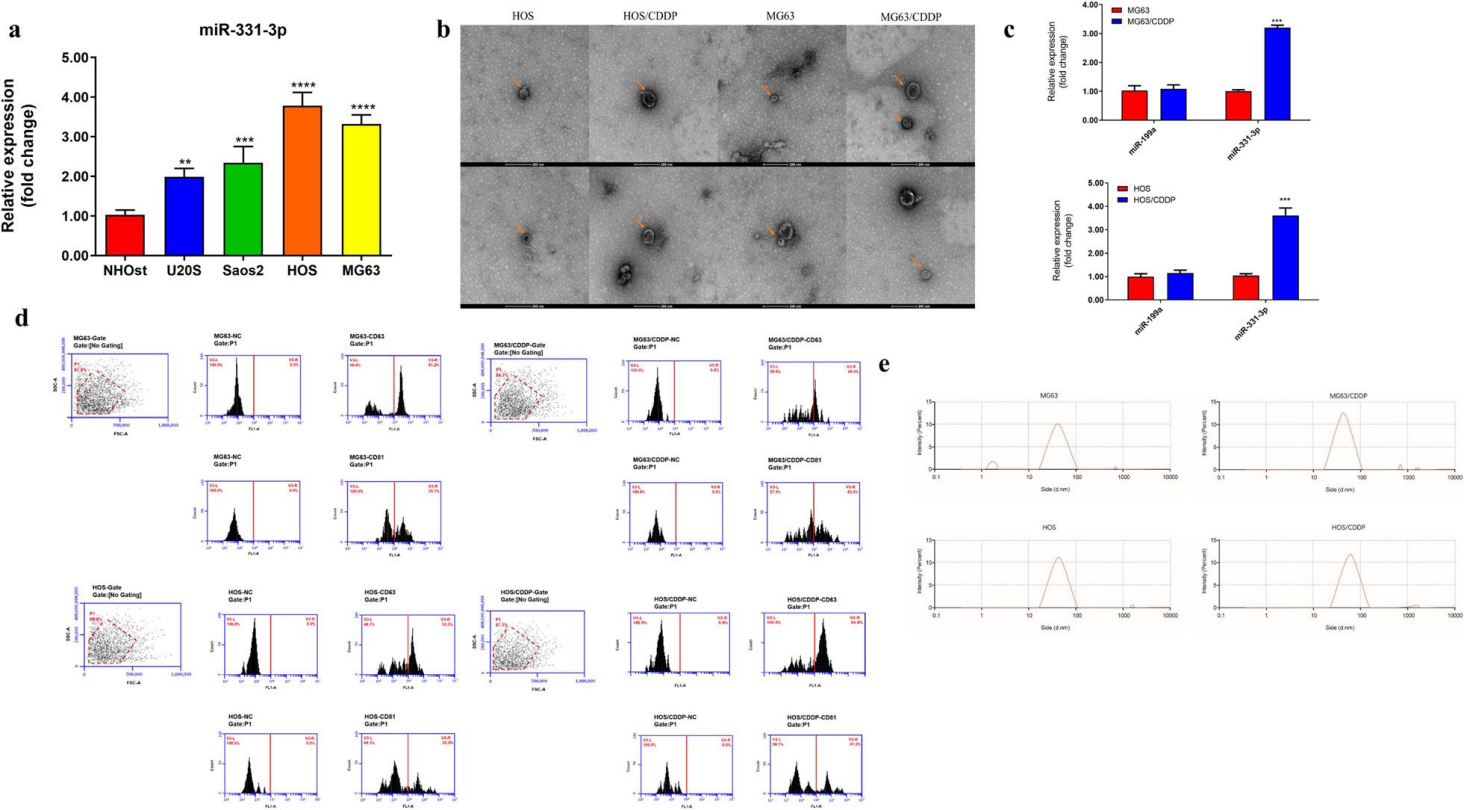
**a** The expression of miR-331-3p in osteosarcoma cell lines and the osteoblastic cell line. **b** Transmission electron microscopy image of exosomes from MG63 and HOS cells (the orange arrow indicates exosomes). **c** The expression of miR-331-3p and miR-199a in exosomes from non-resistant cells (MG63 and HOS) and CDDP-resistant cells (MG63/CDDP and HOS/CDDP). **d** Flow cytometry was used to analyze the expression of CD63 and CD81 surface proteins in the supernatant exosomes. **e** Granulometric analysis of the exosomes. *P < 0.05, **P < 0.01, ***P < 0.001 versus the osteoblastic cell line (NHOst). CDDP, cisplatin

The expression of miR-331-3p in osteosarcoma cell lines was higher in the U20S, Saos2, HOS, and MG63 cell lines than in the NHOst cell line. The expression of miR-331-3p was higher in the MG63 and HOS cell lines than in the other osteosarcoma cell lines (Fig. 1a). Therefore, the MG63 and HOS cell lines were used for the subsequent experiments.

### The exosomes were successfully observed by electron microscopy

Electron microscopy images of exo from MG63, HOS, MG63/CDDP, and HOS/CDDP cells are shown in Fig. 1b. The exo showed a typical “disk-cup” and double-layer membrane structure under transmission electron microscopy (60,000 × magnification).

### miR-331-3p levels in the exosomes of OSCs

The level of miR-331-3p in exo from MG63/CDDPs and HOS/CDDPs was higher than in MG63 and HOS cells, respectively, while there were no differences in miR-199a levels between the MG63/CDDP and MG63 exo (Fig. 1c).

### The exosomes were confirmed through the expression of CD63 and CD81

Flow cytometry was used to analyze the expression of CD63 and CD81 surface proteins in the supernatant exo of the OSCs and CDDP-resistant OSCs (Fig. 1d). The positive rates of CD63 in the supernatant exo of the MG63 and MG63/CDDP cells were 51.2% and 40.4%, respectively. The positive rates of CD81 were 35.1% and 42.5%, respectively. The positive rates of CD63 in the supernatant exo of the HOS and HOS/CDDP cells were 53.3% and 64.8%, respectively. The positive rates of CD81 were 30.5 and 42.5%, respectively. Therefore, the exo nature of the particles was confirmed.

### Granulometric analysis

As shown in Fig. 1e and Table 2, the particle distribution coefficients of the detected samples were between 0.09 and 0.8 in the OSCs and OSC/CDDPs, which showed that the dispersion of the collected exo was moderate and the confidence of the detection results was high. The particle sizes of the MG63 and MG63/CDDP samples ranged from 30 to 150 nm, accounting for 90.2% and 92.1%, respectively. The particle sizes of the HOS and HOS/CDDP samples ranged from 30 to 150 nm, accounting for 94.6% and 93.7%, respectively. These characteristics are consistent with those of exo [24].

**Table 2:** Granulometric analysis of the exosomes

Cell type	Distribution coefficient	Particle size (nm)	Percentage (%)
MG63	0.09–0.8	30–150	90.2
MG63/CDDP	0.09–0.8	30–150	92.1
HOS	0.09–0.8	30–150	94.6
HOS/CDDP	0.09–0.8	30–150	93.7

### Exosomes can be absorbed by MG63 and HOS cells

**Fig. 2:**
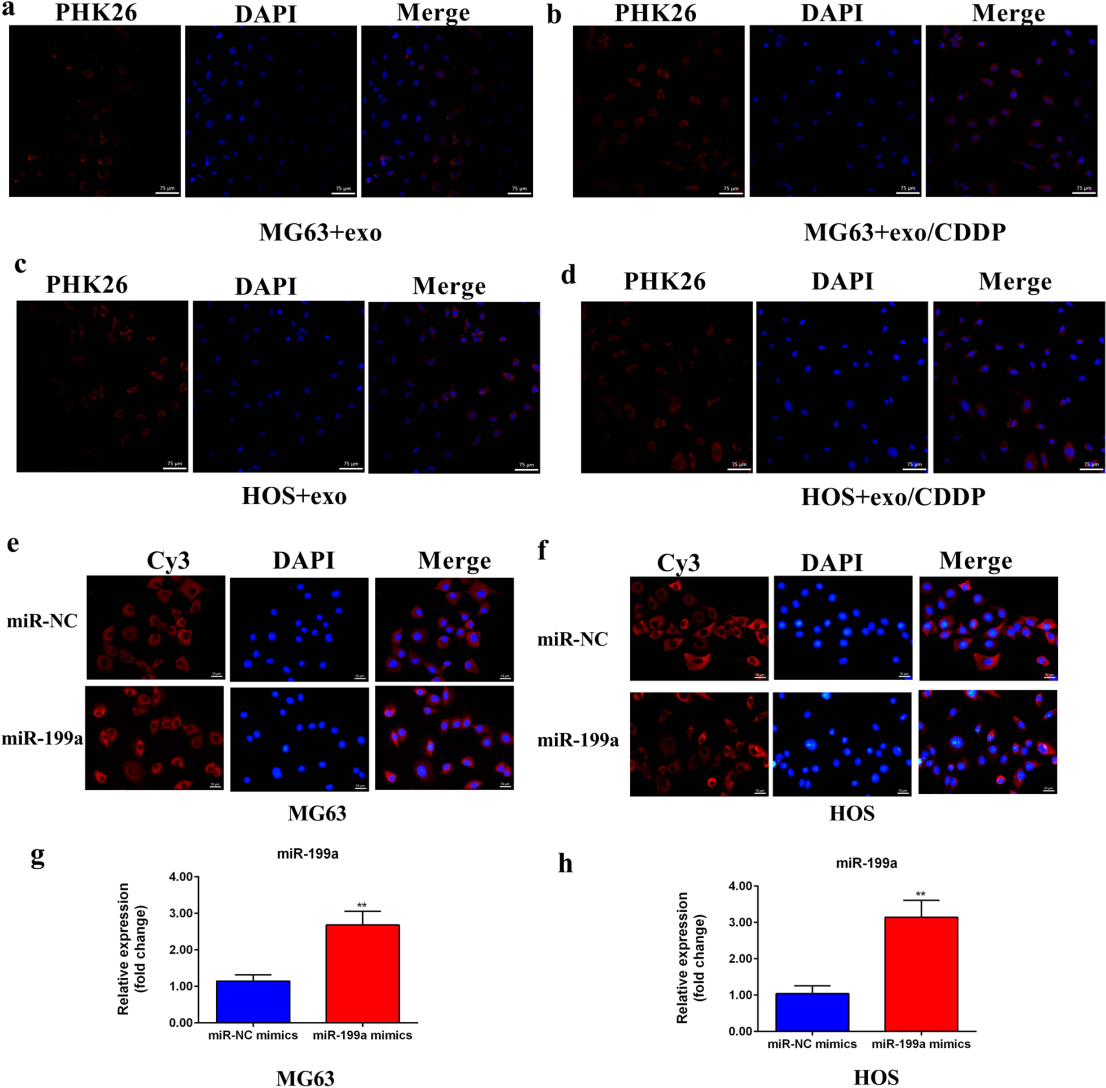
**a** Fluorescence image of PKH26-labeled MG63 cell-derived exosomes co-cultured with MG63 cells. **b** Fluorescence image of PKH26-labeled MG63/CDDP cell-derived exosomes co-cultured with MG63 cells. **c** Fluorescence image of PKH26-labeled HOS cell-derived exosomes co-cultured with HOS cells. **d** Fluorescence image of PKH26-labeled HOS/CDDP cell-derived exosomes co-cultured with HOS cells (**e** and **g**). The fluorescence of Cy3 in MG63 cells was observed under a confocal microscope. The mRNA expression level of miR-199a in MG63 cells in the lower compartment of the Transwell chamber (**f** and **h**). The fluorescence of Cy3 in HOS cells was observed under a confocal microscope. The mRNA expression level of miR-199a in HOS cells in the lower compartment of the Transwell chamber. **P < 0.01 versus the control group. CDDP, cisplatin

PHK26-labeled exo from MG63/CDDP and HOS/CDDP cells could be absorbed by MG63 and HOS cells (Fig. 2a–d).

Since exo usually carry miRNAs and there were no expression differences of miR-199a between OSCs and OSCs/CDDP, miR-199a was used to identify the transmission of miRNA between OSC/CDDP and OSC. According to the Transwell experiment, PHK26-labeled exo and exo/CDDP could be internalized by HOS cells (Fig. 2c–d). The qRT-PCR analyses showed that the exo could be transferred into MG63 and HOS cells to overexpress miR-199a (Fig. 2e–h). Hence, miRNA could be transported from OSC/CDDP into OSC.

### miRNAs carried by exo from CDDP-resistant cells could confer CDDP resistance to previously non-resistant cells

**Fig. 3:**
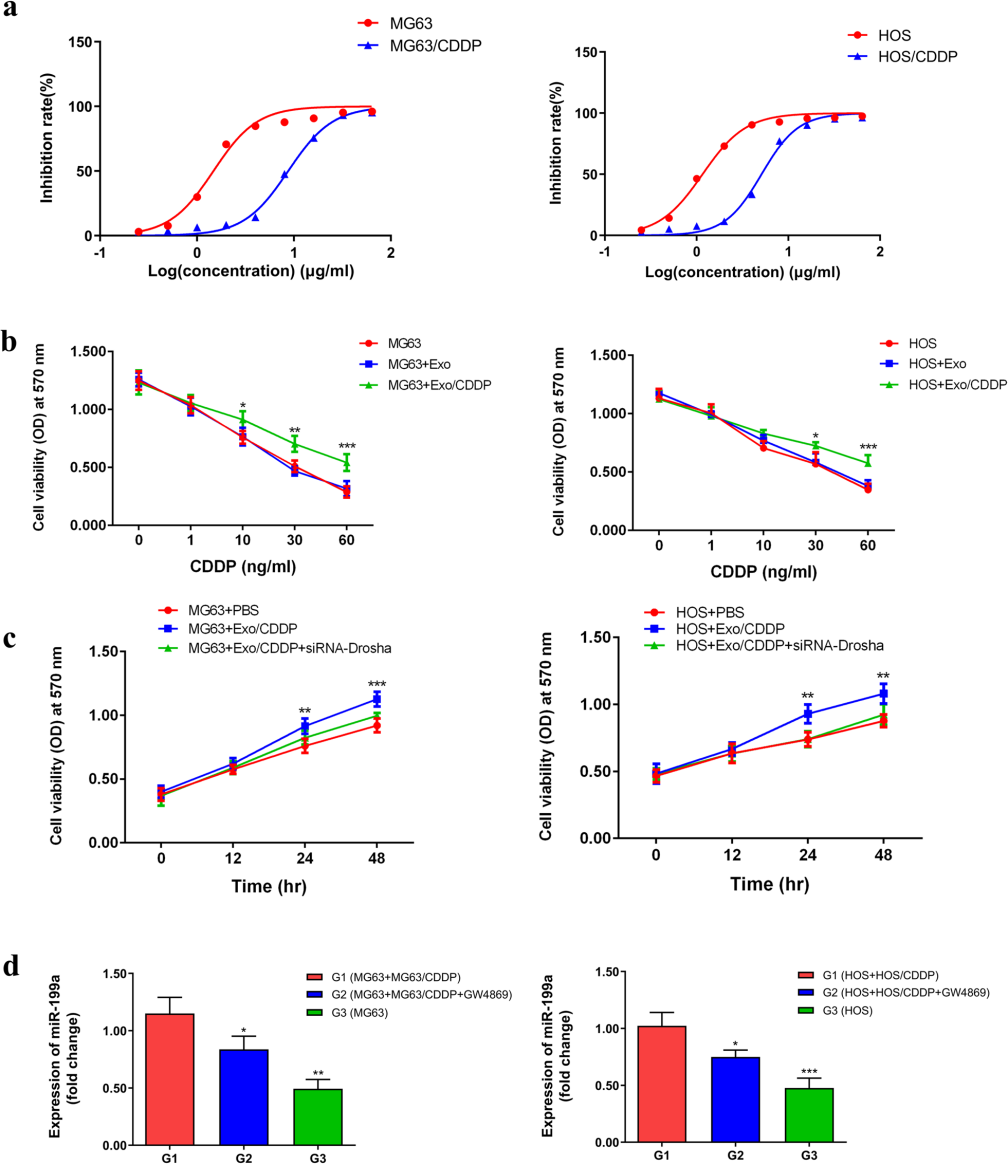
**a** The inhibition curve of CDDP on drug-resistant and sensitive cells of MG63 and HOS. **b** The cell viability of the MG63, MG63 + Exo, and MG63 + Exo/CDDP groups (left) and the HOS, HOS + Exo, and HOS + Exo/CDDP groups (right) in the presence of 10, 30, and 60 ng/mL of cisplatin according to the MTT assay. **c** The cell viability in the MG63, MG63 + Exo/CDDP, and MG63 + Exo/CDDP + siRNA-Drosha (left) and HOS, HOS + Exo, HOS + Exo/CDDP, and HOS + Exo/CDDP + siRNA-Drosha (right) at 0, 12, 24, and 48 h. *P < 0.05, **P < 0.01, ***P < 0.001 versus the control group. **d** The expression of miR-199a in the MG63/CDDP, MG63 + Exo/CDDP, and MG63 + Exo/CDDP + GW4869 groups (left) and the HOS/CDDP, HOS + Exo/CDDP and HOS + Exo/CDDP + GW4869 groups (right). **P < 0.001 versus the MG63/CDDP and HOS/CDDP control groups. CDDP, cisplatin; Exo, exosomes; MTT, 3-[4, 5-dimethylthiazol-2-yl]-2,5-diphenyltetrazolium bromide; siRNA, small interfering RNA

The inhibition curve of CDDP on drug-resistant and sensitive cells of MG63 and HOS was shown in Fig. 3a. The IC50 (half maximal inhibitory concentration) of MG63 and MG63/CDDP were 1.48 μg/mL and 8.71 μg/mL, respectively. The IC50 of HOS and HOS/CDDP were 1.16 μg/mL and 5.10 μg/mL, respectively. In the presence of CDDP at 10, 30, and 60 ng/mL, the proliferation of MG63 and HOS cells was enhanced after treatment with exo isolated from their CDDP-resistant cell counterparts, according to the MTT assay (Fig. 3b). In order to further clarify whether the emergence of OSC resistance is due to exosome-carrying miRNAs, we treated OSC/CDDP with siRNA-Drosha. Exo from MG63/CDDP and HOS/CDDP transfected by siRNA-Drosha could not enhance the proliferation of MG63 and HOS cells treated with CDDP (Fig. 3c). These results suggest that the miRNAs carried by exo from CDDP-resistant cells could confer CDDP resistance to previously non-resistant cells.

OSCs were co-incubated with OSCs/CDPP to see if the additional administration of GW4869 (an exo formation inhibitor) inhibits the transfer of exo and miRNA. The expression of miR-199a in OSCs co-incubated with CDDP-resistant cells was (G1) higher than in the OSCs group (G3). OSCs co-incubated with OSCs/CDPP, which treated by GW4869 (G2) was lower than that in G1 (Fig. 3d). Hence, GW4869 inhibits exo formation and miR-199a transfer. These results suggest that exo from OSC/CDDP could transfer miRNAs. GW4869 inhibited exo formation and thus was unable to transport miRNA. miR-199 was reduced after inhibition of exo release using GW4689, indicating that it is the exo that transports the miRNA from the resistant cells to the recipient cells. miR-199a was chosen because it was not differentially expressed in the two cells. Therefore, after blocking exo, miRNA from drug-resistant cells could not enter the recipient cells.

### miR-331-3p carried by exosomes could promote autophagy to induce drug resistance in osteosarcoma

**Fig. 4:**
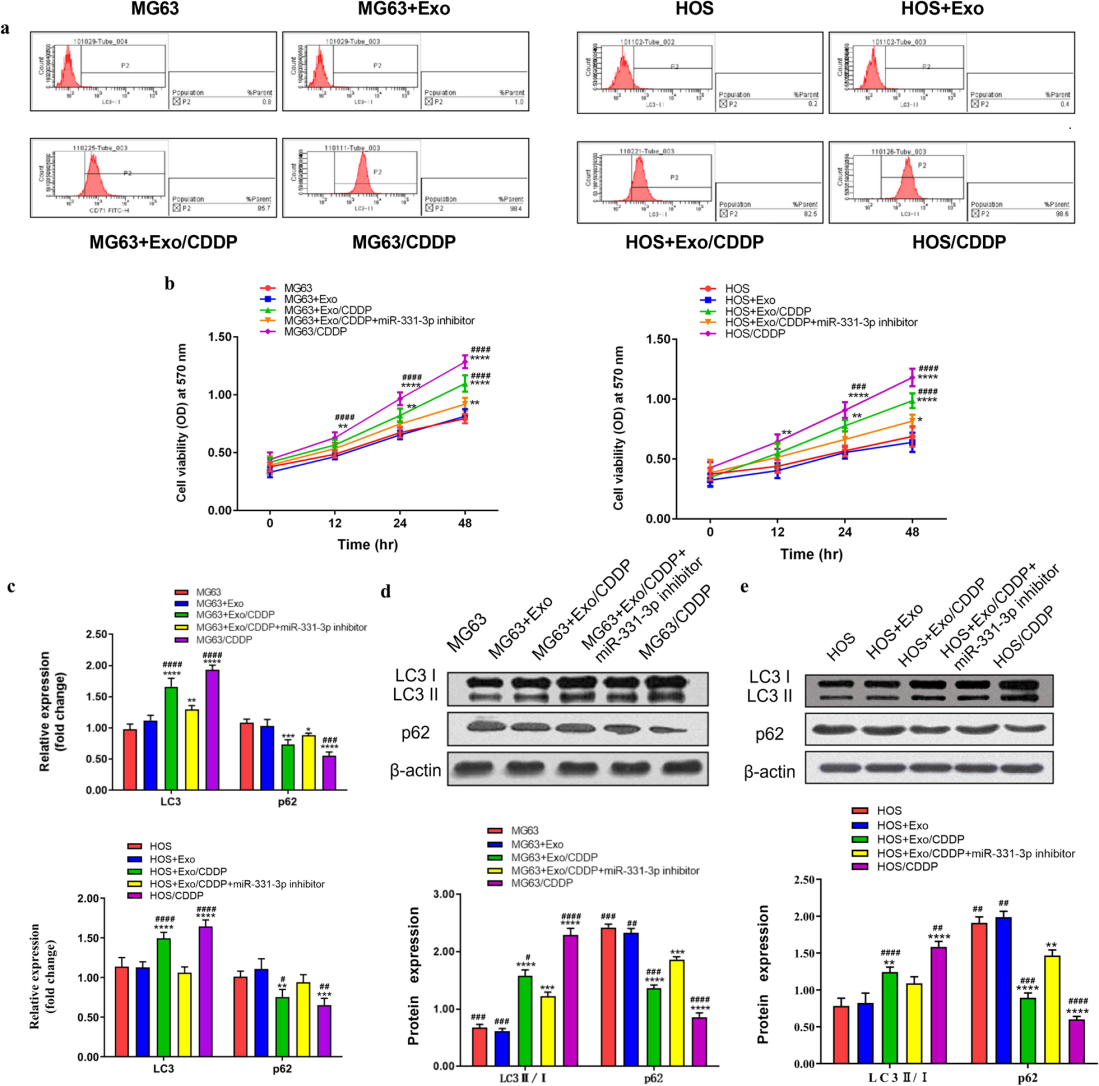
**a** LC3 flow cytometry staining to detect autophagy in the MG63, MG63 + Exo, MG63 + Exo/CDDP, and MG63/CDDP groups (left) and the HOS, HOS + Exo, HOS + Exo/CDDP, and HOS/CDDP groups (right). **b** The cell viability of the MG63, MG63 + Exo, MG63 + Exo/CDDP, MG63 + Exo/CDDP + miR-331-3p inhibitor, and MG63/CDDP groups after being treated with CDDP for 12, 24, and 48 h. The right part showed the results for HOS cells. **c** The mRNA expression of LC3 and p62 in the MG63, MG63 + Exo, MG63 + Exo/CDDP, MG63 + Exo/CDDP + miR-331-3p inhibitor, and MG63/CDDP groups after treatment using CDDP for 24 h (upper for MG63 and lower for HOS). **d** The protein levels of LC3 and p62 in the MG63, MG63 + Exo, MG63 + Exo/CDDP, MG63 + Exo/CDDP + miR-331-3p inhibitor, and MG63/CDDP groups after treatment using CDDP for 24 h. **e** The same for the HOS cells. **P < 0.01, ***P < 0.001, ****P < 0.0001 versus the MG63, HOS control group. ^#^*P* < 0.05, ^##^P < 0.01, ^###^*P* < 0.001, ^####^*P* < 0.0001 versus the MG63, HOS + exo/CDDP + miR-331 inhibitor group. CDDP, cisplatin; Exo, exosomes; LC3, microtubule-associated protein 1 light chain 3 alpha

According to the LC3 flow cytometry, exo from CDDP-resistant OSCs could induce autophagy of OSCs (MG63 and HOS). The LC3 levels were higher in CDDP-resistant OSCs than in OSCs (MG63 and HOS); in the P2 region, the percentage of LC3 expression was 0.8% and 1.0% for MG63 and MG63 + exo, and 85.7% and 98.4% for MG63 + exo/CDDP and MG63/CDDP; the percentage of LC3 expression was 0.2% and 0.4% for HOS and HOS + exo, and the percentage of HOS + exo/CDDP and HOS/CDDP LC3 expression accounted for 82.5% and 98.6% (Fig. 4a).

Since the level of miR-331-3p in the exo from MG63/CDDP cells is higher than in MG63 cells, and miRNAs from drug-resistant cells cause drug resistance in non-drug-resistant cells, we speculate that miR-331-3p may be one of them. To further clarify the role of miR-331-3p, we transfected OSC/CDDP with an inhibitor of miR-331-3p.

In the presence of CDDP, the cell proliferation of the MG63/CDDP group was the highest, and the cell proliferation of MG63 could be improved after treatment by exo from MG63/CDDPs. The cell proliferation of MG63s treated by exo from MG63/CDDP cells transfected by a miR-331-3p inhibitor was reduced compared with that of MG63 cells treated by exo from MG63/CDDP without transfection of the miR-331-3p inhibitor. HOS and MG63 cells showed similar results (Fig. 4b). According to the results of qRT-PCR and western blotting, the expression of LC3 increased in MG63 cells after treatment by exo from MG63/CDDP but decreased in MG63 cells treated by exo from MG63/CDDP cells transfected with the miR-331-3p inhibitor. In addition, the expression of p62 was reduced in MG63 after treatment by exo from MG63/CDDP cells but was increased in MG63 cells treated by exo from MG63/CDDP cells transfected with the miR-331-3p inhibitor. miR-331-3p inhibitor could reduce autophagy compared with MG63/CDDP and MG63 treated with exo/CDDP group. HOS cells and MG63 cells showed similar results (Fig. 4c–e). Therefore, miR-331-3p carried by exo from drug-resistant cells could promote autophagy to induce drug resistance in osteosarcoma cells.

## Discussion

This study aimed to explore the mechanism of exosomal miR-331-3p in chemoresistance in osteosarcoma. The results suggest that exo secreted from chemoresistant osteosarcoma cells promote drug resistance through miR-331-3p and autophagy. Hence, inhibition of miR-331-3p could alleviate drug resistance in osteosarcoma.

In this study, the transmission role of miRNA-exo was investigated in drug resistance in osteosarcoma. It has been reported that osteosarcoma chemoresistant cells can transmit multiple drug-resistant gene phenotypes into non-drug-resistant cells via exo, causing non-drug-resistant OSCs to develop drug resistance [17]. Besides, exo could carry DNAs, RNAs, lipids, and proteins for cellular communication in tumor development, and it has been widely investigated in bone sarcomas [25, 26]. Exosomes participate in numerous physiological and pathological processes via intercellular substance exchange and signaling [27]. The results of the present study are consistent with the previous study. Indeed, the non-resistant OSCs could internalize the exo isolated from CDDP-resistant OSCs, but non-resistant OSCs exposed to exo from CDDP-resistant OSCs developed CDDP resistance. Furthermore, chemoresistance could be prevented by inhibiting the production of exo or suppressing miRNAs. Non-code RNA has been widely explored. It has been reported that CircDOCK1 promotes the tumorigenesis and cisplatin resistance of osteogenic sarcoma via the miR-339-3p/IGF1R axis [28]. Furthermore, previous studies focused on the cell-derived exo that regulate the proliferation, invasion, and chemoresistance of OSCs. BMSCs-derived exo miR-208a promotes the proliferation and metastasis of OSCs [29]. Another study found that the exo secreted by metastatic OSCs could deliver miR-675 to regulate the bioactivity of osteosarcoma by targeting Calnexin-1 [17]. Wang et al. [30] reported that the exo secreted by tumor-related fibroblasts could transmit miR-1228 into OSCs to promote the proliferation and invasion of OSCs by targeting SCAI (which encodes a suppressor of cancer cell invasion). Thus, miRNA-containing exo are an effective transmission route for various factors affecting the biological behavior of OSCs, including drug resistance.

In the present study, the involvement of miR-331-3p was identified as being involved in the transmission of drug resistance in OSCs. It has been reported that miR-331-3p could inhibit the proliferation and migration of colon cancer cells by targeting NRP2 (Neuropilin 2) [30]. Another study showed that circ-0001649 sponges and inhibits the function of miR-331-3p, suppressing non-small cell lung cancer [31]. A high serum miR-331-3p expression was considered a high-risk factor for esophageal cancer recurrence [32]. A study on miR-331 and chemotherapy in leukemia reported that an increased expression of miR-331 might lead to poor treatment efficacy and low survival rates [33]. Therefore, these previous studies support that miR-331-3p can cause tumor recurrence and poor prognosis. Still, the previous studies also highlight that the expression profile and regulatory roles of miR-331-3p are different across different tumor types. Bi et al. [34] reported that miR-331-3p could suppress osteosarcoma progression by targeting MGAT1, involving the Bcl-2/Bax and Ent/β-Catenin pathways. Zu et al. [35] reported that miR-331-3p overexpression inhibited osteosarcoma cell proliferation, metastasis, and invasion by targeting the SOCS1/JAK2/STAT3 pathway. Still, no relevant reports have been published regarding chemoresistance in osteosarcoma and miR-331-3p. In the present study, the expression of miR-331-3p in chemoresistant OSCs was higher than in non-resistant OSCs, suggesting that miR-331-3p participates in the chemoresistance or aggressiveness of osteosarcoma. Most importantly, the present study showed that miR-331-3p can be transmitted among cells via exo, and that the exo-carried miR-331-3p can induce chemoresistance in previously non-resistant cells. Still, the exact mechanisms of this chemoresistance due to miR-331-3p remains elusive in osteosarcoma, and, as highlighted above, the mechanisms might differ among cancer types. Nevertheless, so far, miR-331-3p is upregulated in pancreatic cancer cells, where it induces resistance to gemcitabine by activating the Wnt/β-catenin signaling through ST7L [36]. miR-331-3p has been shown to participate in the epithelial-to-mesenchymal transition [37–39], and cells that underwent that transition can be more resistant to chemotherapy [40]. Since the Wnt/β-catenin signaling pathway is also involved in chemoresistance in osteosarcoma, the involvement of miR-331-3p and Wnt/β-catenin signaling in the chemoresistance of OSCs should be investigated in future studies. Therefore, the miR-331-3p-related chemoresistance observed here, and the miR-331-3p-inhibited osteosarcoma aggressiveness in previous studies [34, 35] could be context-dependent. Still, studies are needed since the same pathways appear to be involved in both processes.

In this study, exo from chemoresistant OSCs could induce autophagy of OSCs. Programmed cell death including ferroptosis, necroptosis and pyroptosis, governed by a diverse array of genes, serves as a pivotal mechanism in the progression and maturation of organisms. Additionally, it is essential for the preservation of tissue and organ equilibrium and contributes to numerous pathological phenomena [41]. Autophagy is an important pathway involved in the drug resistance of cancer cells [18]. In the present study, miR-331-3p could cause autophagy of OSCs, while the inhibition of miR-331-3p suppressed the autophagy of OSCs. Inhibition of miR-331-3p and miR-9-5p ameliorated Alzheimer’s disease by enhancing autophagy [42]. Thus, it could be hypothesized that exo miR-331-3p induced autophagy and increased the cell vitality and chemoresistance of OSCs. Still, autophagy is a complex process that several factors can influence in vivo, and additional studies are still necessary to examine the effects and regulation of autophagy in OSCs. Still, it is increasingly being recognized that autophagy is involved in the chemoresistance of osteosarcoma [43], and thus modulating autophagy could help improve the prognosis of the patients through a better response to treatments. Of course, this study has limitations. It focused on a single miR-331-3p, while it is known that exo carry multiple miRNAs that can act synergistically or additively on the target cells. The exact molecular mechanisms involved in miR-331-3p-related chemoresistance were not investigated. Only cells were investigated, and future studies should include xenografts in nude mice.

## Conclusions

Exo secreted from chemoresistant osteosarcoma cells promote drug resistance through miR-331-3p and autophagy. Inhibition of miR-331-3p could be used to alleviate drug resistance in osteosarcoma.

## Data Availability

All data generated or analyzed during this study are included in this published article.

## Abbreviations

BMSCs: Bone marrow mesenchymal stem cells
CDDP: Cisplatin
exo: Exosome
miRNA: MicroRNA
mRNA: Messenger RNA
OSC: Osteosarcoma cell
